# Heterologous expression of the atypical tetracycline chelocardin reveals the full set of genes required for its biosynthesis

**DOI:** 10.1186/s12934-020-01495-x

**Published:** 2020-12-19

**Authors:** Tadeja Lukežič, Špela Pikl, Nestor Zaburannyi, Maja Remškar, Hrvoje Petković, Rolf Müller

**Affiliations:** 1 Department of Microbial Natural Products, Helmholtz Institute for Pharmaceutical Research Saarland (HIPS)-Helmholtz Centre for Infection Research (HZI), and Department of Pharmacy, Saarland University Campus, Campus E8.1, 66123 Saarbrücken, Germany; 2 German Center for Infection Research (DZIF), Partner Site Hannover-Braunschweig, 38124 Braunschweig, Germany; 3 Department of Food Science and Technology, Biotechnical Faculty, University of Ljubljana, Jamnikarjeva 101, 1000 Ljubljana, Slovenia; 4 Present Address: National Institute of Biology, Večna pot 111, 1000 Ljubljana, Slovenia

**Keywords:** Antibiotics, Polyketide, Heterologous expression, Actinobacteria, Natural product biosynthesis, Tetracyclines, Chelocardin

## Abstract

**Background:**

Chelocardin (CHD) exhibits a broad-spectrum antibiotic activity and showed promising results in a small phase II clinical study conducted on patients with urinary tract infections. Importantly, CHD was shown to be active also against tetracycline-resistant Gram-negative pathogens, which is gaining even more importance in today’s antibiotic crisis. We have demonstrated that modifications of CHD through genetic engineering of its producer, the actinomycete *Amycolatopsis sulphurea*, are not only possible but yielded even more potent antibiotics than CHD itself, like 2-carboxamido-2-deacetyl-chelocardin (CD-CHD), which is currently in preclinical evaluation. *A. sulphurea* is difficult to genetically manipulate and therefore manipulation of the *chd* biosynthetic gene cluster in a genetically amenable heterologous host would be of high importance for further drug-discovery efforts.

**Results:**

We report heterologous expression of the CHD biosynthetic gene cluster in the model organism *Streptomyces albus* del14 strain. Unexpectedly, we found that the originally defined CHD gene cluster fails to provide all genes required for CHD formation, including an additional cyclase and two regulatory genes. Overexpression of the putative pathway-specific streptomyces antibiotic regulatory protein *chdB* in *A. sulphurea* resulted in an increase of both, CHD and CD-CHD production. Applying a metabolic-engineering approach, it was also possible to generate the potent CHD analogue, CD-CHD in *S. albus*. Finally, an additional yield increase was achieved in *S. albus* del14 by in-trans overexpression of the *chdR* exporter gene, which provides resistance to CHD and CDCHD.

**Conclusions:**

We identified previously unknown genes in the CHD cluster, which were shown to be essential for chelocardin biosynthesis by expression of the full biosynthetic gene cluster in *S. albus* as heterologous host. When comparing to oxytetracycline biosynthesis, we observed that the CHD gene cluster contains additional enzymes not found in gene clusters encoding the biosynthesis of typical tetracyclines (such as oxytetracycline). This finding probably explains the different chemistries and modes of action, which make CHD/CD-CHD valuable lead structures for clinical candidates. Even though the CHD genes are derived from a rare actinomycete *A. sulphurea*, the yield of CHD in the heterologous host was very good. The corrected nucleotide sequence of the CHD gene cluster now contains all gene products required for the production of CHD in a genetically amenable heterologous host, thus opening new possibilities towards production of novel and potent tetracycline analogues with a new mode of action.

## Background

**Fig. 1:**
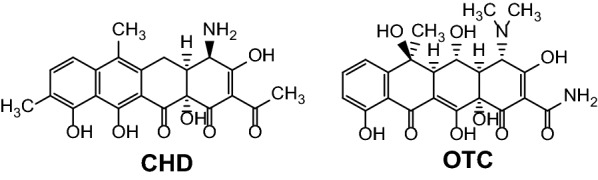
Structures of CHD and OTC

Facing increasingly spreading antimicrobial resistance and a severe shortage of novel anti-infectives, one of the strategies to develop new antibiotics is the revival and chemical optimisation of validated chemical scaffolds, such as the tetracyclines (TCs). We previously reported cloning of the biosynthetic gene cluster (BGC) encoding the biosynthesis of the atypical TC, chelocardin (CHD) [1]. CHD belongs biosynthetically to the group of aromatic polyketides which are biosynthesized through decarboxylative condensation of malonate building blocks and subsequent directed cyclization of the nascent precursor polyketide chain thereby resulting in a tetracyclic scaffold [2]. The CHD scaffold itself is further chemically decorated by so called post-PKS enzymes such as oxygenases, methyltransferases and aminotransferases [3], yielding structurally relatively small, but important differences in the structure of CHD compared to the typical TCs. These modifications include a different aromatization pattern of the TC backbone, an acetyl instead of a carboxamido group at carbon 2 [C2], a primary instead of tertiary amine at C4 in opposite configuration, and an additional methyl group at carbon C9 (Fig. 1). While it is apparent that these differences are reflected in a different mode of action of CHD compared to typical TCs [4, 5], the molecular target of CHD has not been identified yet. We have demonstrated that it is possible to carry out modifications of CHD through genetic engineering of its producer, the actinomycete *Amycolatopsis sulphurea*. However, *A. sulphurea* is difficult to genetically manipulate and therefore engineering of the *chd* biosynthetic gene cluster in a genetically amenable heterologous host would be of high importance for further drug-discovery efforts.

CHD has caught our attention as it exhibits a broad-spectrum antibiotic activity and showed promising results in a small phase II clinical study conducted on patients with urinary tract infections caused by Gram-negative pathogens in 1977 [6]. CHD was shown to be active also against TC-resistant pathogens, including Gram-negative bacteria [7], which is gaining even more importance in today’s antibiotic crisis.

In the recent years, we have demonstrated that modifications of CHD through genetic engineering of its producer, the actinomycete *A. sulphurea*, are not only possible but yielded even more potent antibiotics than CHD itself, like 2-carboxamido-2-deacetyl-chelocardin (CDCHD) [8]. Our work also provided an understanding of the structure–activity relationship of CHD [9]. Furthermore, CDCHD was semi-synthetically modified [9, 10] with the aim to optimize the potent CHD derivative even further.

Here, we report heterologous expression of the CHD biosynthetic gene cluster in the model organism *Streptomyces albus* del14 strain [11]. Unexpectedly we here demonstrate that the originally identified CHD BGC [1] fails to provide all genes required for CHD formation, including an additional cyclase and regulatory genes. When comparing to oxytetracycline (OTC) BGC, we have observed that CHD BGC can actually contain genes which are generally not found in typical tetracycline gene cluster. By successful heterologous expression of CHD in a *Streptomyces* host, we confirmed that the cloned CHD BGC from this work contains all gene products required for regulation, cyclization and formation of the TC backbone of CHD, thus enabling and extending biosynthetic engineering efforts, which were shown previously to be very promising for generating new CHD analogues, including CDCHD with potent bioactivity. Considering that gene-manipulation in the native producer *A. sulphurea* is technically demanding, heterologous expression in a genetically amenable actinomycete cell factory would be an important advantage when aiming to generate novel CHD analogues by biosynthetic engineering approaches.

## Results and discussion

### Chelocardin biosynthetic gene cluster

**Fig. 2:**
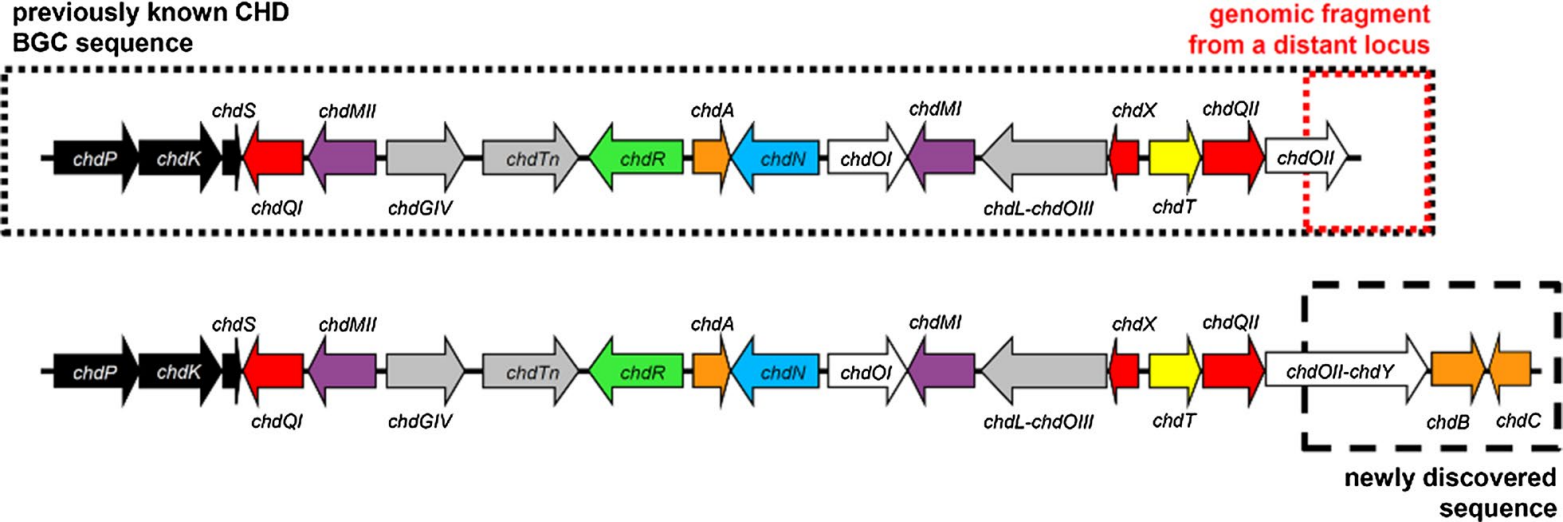
Gene cluster of chelocardin showing newly-identified genes involved in chelocardin biosynthesis

When comparing the published nucleotide sequence of CHD BGC [1], which was based on sequencing of a cosmid selected from a cosmid library, to the *A. sulphurea* NRRL 2822 whole-genome sequence established in the scope of this work, we found that the original sequence of the CHD BGC was misassembled. It turned out that the original cosmid containing the CHD BGC was chimeric and composed of two genomically unlinked fragments (Fig. 2). Correspondingly, we identified an additional DNA fragment containing genes involved in CHD biosynthesis; one additional putative cyclase gene homologue for the biosynthesis of CHD, and two additional putative regulatory genes (Fig. 2). The new biosynthetic gene was designated as *chdY* encoding a putative second ring cyclase protein, a homologue to OxyN from OTC biosynthesis [12]. We also identified a likely mistake in the original CHD BGC sequence, where the C-terminal part of *chdOII* was not correctly annotated. After correction, the C-terminal part of the *chdOII* DNA sequence revealed even higher similarity to the *oxyL* encoding the oxygenase from OTC BGC involved in TC scaffold formation in OTC biosynthesis (Fig. 2) [12]. These corrections were introduced into the current GenBank Accession Number KC870000.

Interestingly, in the CHD BGC, ChdOII and ChdY (Table 1) are encoded as one single polypeptide, thus forming a bifunctional protein. In contrast, two separate ORFs encoding OxyL and OxyN (homologues of ChdOII and ChdY, respectively) are present in OTC BGC. Analogously, this is also observed for *chdL* and *chdOIII* (Table 1) nucleotide sequences from CHD BGC, which also yield one single polypeptide. Similarly, *chdL* and *chdOIII* homologues from OTC BGC, *oxyH* and *oxyG*, respectively, are translationally coupled (Table 1).

**Table 1:** Proposed function of gene homologues identified in the CHD BGC

Gene	Size (AA)	Putative function	Protein homologue (GenBank Accession Number)	Identity/similarity (%)
*chdP*	432	Ketosynthase alpha	OxyA (P43678.2)	78/85
*chdK*	418	Ketosynthase beta	OxyB (AAZ78326.1)	71/82
*chdS*	88	Acyl carrier protein	OxyC (P43677.1)	62/77
*chdQI*	302	Aromatase/cyclase	OxyK (AAZ78334.2)	32/48
*chdMII*	343	Methyltransferase	CmmMII (CAE17532.1)	45/61
*chdGIV*	399	Glycosyltransferase	CmmGIV (CAE17547.1)	39/51
*chdTn*	511	Transposase	InsG (P03835.1)	25/40
*chdR*	481	Exporter	EmrB (P9WG88.1)	33/54
*chdA*	190	Transcriptional regulator	TetR (5MRU_A)	43/56
*chdN*	448	Aminotransferase	Msat (1WST_A)	31/47
*chdOI*	404	Oxygenase	OxyE (AAZ78329.1)	66/78
*chdMI*	341	Methyltransferase	OxyF (AAZ78330.1)	66/76
*chdL-chdOIII*	643	Acyl-CoA ligase/oxygenase	OxyH (ELQ83297.1)	56/69
*chdOIII*	96	Oxygenase	OxyG (AAZ78331.1)	63/76
*chdX*	150	Cyclase	OxyI (AAZ78332.2)	64/74
*chdT*	262	Ketoreductase	OxyJ (AAZ78333.1)	76/85
*chdQII*	315	Aromatase/cyclase	OxyK (AAZ78334.2)	58/67
*chdOII-chdY*	806	Oxygenase/cyclase	OxyL (AAZ78335.1)	59/69
*chdY*	256	Cyclase	OxyN (AAZ78337.1)	73/80
*chdB*	258	Transcriptional activator (SARP family)	OtcR (AJO26937.1)	45/63
*chdC*	212	Transcriptional activator (LuxR family)	OtcG (ACM67367.1)	35/56

Finally, bioinformatic analysis of the DNA sequence downstream of the newly-identified putative cyclase gene *chdY* revealed two regulatory genes, *chdB* and *chdC*, encoding SARP and LuxR type regulators, respectively (Table 1). *chdB* and *chdC* homologues, *otcR* and *otcG*, respectively, are also identified in OTC and chlortetracycline BGCs in *S. rimosus* and *S. aureofaciens,* respectively (Fig. 2) [13, 14].

### Proposed CHD biosynthetic pathway

**Fig. 3:**
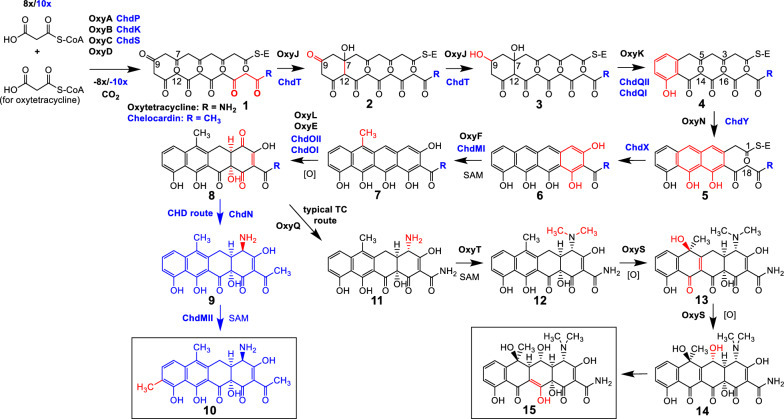
Schematic presentation of the proposed OTC and CHD biosynthetic pathways

Considering the additional part of CHD BGC, we repeated the analysis of the CHD BGC and re-assigned the putative biosynthetic steps (Fig. 3) in comparison to the biosynthetic pathway of OTC [2, 12]. We focused our study on the putative gene homologues involved in formation of the basic TC scaffold. Thus, when comparing early stages in OTC and CHD biosynthesis, gene homologues can be identified in both BGCs, which are necessary for the formation of early putative biosynthetic intermediates such as 4-keto-ATC in OTC pathway (**8**, Fig. 3), strongly resembling the putative CHD precursor, 4-keto-9-desmethyl-CHD (**8**, Fig. 3). These two intermediates differ only in the moiety attached at the C2 position, as a consequence of incorporation of a different starter unit. However, it is important to mention a known congener in OTC biosynthesis (**8**, Fig. 3) leading to a well-known impurity, 2-acetyl-2-decarboxamido-OTC (AD-OTC, [12]), which is presumably primed by the same starter unit as in CHD biosynthesis. Analogously, in CHD biosynthesis, AD-OTC should then be structurally identical to 4-keto-9-desmethyl-CHD (**8**, Fig. 3).

The enzymes putatively involved in CHD biosynthesis are marked in blue colour. Similarly, the possible intermediate structures, belonging exclusively to the CHD biosynthetic pathway branch are labelled in blue colour. Finally, numbering of carbon atoms is according to chemical nomenclature.

The formation of the TC backbone in CHD is catalyzed by the type II minimal polyketide synthase (minimal PKS) genes, consisting of ketosynthase α, ketosynthase β and acyl carrier protein (ACP), designated as ChdP, ChdK and ChdS, respectively. It is expected that the minimal PKS of CHD condenses 10 malonyl-CoA building blocks into an acetate-primed decaketide (**1**, Fig. 3) [1]. The malonyl-CoA:ACP acyltransferase required for the transfer of the extender unit malonyl-CoA to the ACP, was proposed to be shared with fatty acid biosynthesis [15]. Analogously as in OTC biosynthesis, catalyzed by OxyJ [12], initial folding of the growing polyketide chain in CHD is most probably directed by the ketoreductase ChdT, resulting in reduction of the keto group at C9 (**3**, Fig. 3). Closure of 4 rings leading to the formation of the CHD backbone is most likely directed by aromatases/cyclases ChdQI, ChdQII, ChdY and ChdX. The first two are both similar to OxyK, and the latter two are homologous of OxyN and OxyI, respectively [2, 12]. Based on homologies to aromatases/cyclases encoded in other aromatic polyketide BGCs, we propose that the didomain aromatase/cyclases ChdQI and ChdQII are both responsible for formation of first ring D (**4**, Fig. 3), while the monodomain cyclase ChdY is needed for the second ring (C) closure. As is the case in biosynthesis of other aromatic polyketides, formation of third ring (B) could be spontaneous (**5**, Fig. 3) or potentially catalyzed by second ring cyclase as suggested by Hertweck et al. [3, 16]. Possibly, the last ring (A) cyclization is carried out by the cyclase ChdX (**6**, Fig. 3). We have proposed the function of the putative cyclase ChdX based on the comparison with chromomycin and mithramycin biosynthetic pathways [17]. The function of its homologue in OTC biosynthesis, OxyI, on the other hand, has not yet been elucidated and was shown that it is not essential for last ring closure due to the terminal amide moiety, potentially leading to spontaneous cyclization [12, 16]. Interestingly, it was proposed by Pickens et al. that acyl-CoA-ligase SsfL2, OxyH homologue from the OTC BGC, is involved in the formation of the fourth ring of the TC-like compound SF2575 [12, 18]. Indeed, it was later demonstrated that SsfL2 catalyzes an ATP-dependent C1–C18 Claisen condensation to close the fourth ring in the biosynthesis of SF2575 [19, 20]. The nascent TC scaffold undergoes further processing towards the final structure of CHD by so-called post-PKS tailoring reactions. Compared to OTC biosynthesis, the late steps in CHD biosynthesis, namely putative C-9 methylation by ChdMII and transamination of C4 in opposite stereochemistry catalyzed by ChdN, respectively, are the last two steps in CHD biosynthesis and significantly differ from the biosynthesis of typical TCs such as OTC (Fig. 3). The putative C6-methyltransferase ChdMI is a homologue of OxyF from OTC BGC [16], (**7,** Fig. 3). Putative oxygenases ChdOI and ChdOII, homologues of OxyE and OxyL from OTC BGC [21], respectively, carry out hydroxylation of ring A at C4 and C4/C12a, respectively (**8**, Fig. 3). Oxidation at C4 results in formation of a chinone which is reductively transamidated by ChdN (**9**, Fig. 3), a PLP-dependent aminotransferase. Surprisingly, although catalyzing almost identical reactions, ChdN is only distantly related to OxyQ, which is responsible for incorporation of the amino group at C4 in OTC biosynthesis; however, the biosynthesis results in opposite stereochemistry at C4 [12]. The activity of these two divergent aminotransferases represents a branching point between CHD and typical TCs biosynthesis and results in different products. The amino group incorporated into CHD is installed in *R*-configuration, while the one in OTC biosynthesis is in *S*-configuration. In contrast to the more decorated backbone of typical TCs such as OTC, there is only one more tailoring reaction leading to CHD: C9-methylation. This reaction is believed to be catalyzed by ChdMII (**10**, Fig. 3), a homologue of the C9-methyltransferases from chromomycin and mithramycin biosynthesis [17].

Overall, when comparing the biosynthetic pathways of CHD and OTC, a very high degree of similarity between the genes can be observed (Fig. 3). However, a number of genes present in the CHD BGC do not have their counterparts in typical TC BGCs, which particularly relates to the C-9 methyltransferase *chdMII*, the C4-aminotransferase *chdN* and the exporter/resistance gene *chdR*, thus suggesting that the CHD gene cluster evolved by incorporating genes from aureolic acids type II PKS systems such as chromomycin and mithramycin.

When comparing enzymes involved in the early steps of biosynthesis of the basic TC backbone, with exception of starter unit selection and cyclisation of the ring C. The cyclisation of the C-ring in CHD biosynthesis is somehow different, considering that a fully aromatic structure is maintained in CHD biosynthesis (Fig. 1). In OTC biosynthesis, where only D ring is aromatized (Fig. 1), hydroxylation of the positions C5 and C6 is carried out by OxyS and OxyR [22]. CHD BGC does not contain OxyS and OxyR homologues, thus no further hydroxylation occurs in CHD biosynthesis at C5 and C6 positions, and this is probably also a reason that CHD maintains ring C aromatized [1, 23].

The aromatization pattern of C and D rings likely has direct influence on the overall planarity of the backbones of CHD and OTC [24], with implications regarding the different mode of action of CHD. As discussed earlier, an important branching point in the biosynthetic pathways of OTC and CHD is most likely the addition of the amino group with opposite stereochemistry, carried on the putative quinoide intermediate harboring a keto-group at carbon C4 (**8**, Fig. 3). This step also has a very important implication for the activity of CHD [8], in addition to carbon C9 methylation of the CHD backbone, which does not occur in the OTC biosynthesis [8, 12].

### Regulation of CHD biosynthesis and self-resistance

One of the putative regulatory proteins found in CHD BGC (Fig. 2), ChdB, is a close homologue to OtcR in OTC biosynthesis [14]. OtcR is a positive regulatory protein, close homolog of *Streptomyces* antibiotic regulatory protein (SARP) from oxytetracycline, SF2575, dactylocycline and chlortetracycline BGC, OtcR, SsfT1, DacT1, CtcB, respectively as presented on the Additional file 1: Figure S1. OtcR is acting as a pathway-specific activator in OTC biosynthesis in *S. rimosus* and leading to a significant increase in OTC production when overexpressed at the appropriate level [14].

The second putative regulatory protein ChdC, found in the CHD BGC belongs to the LuxR family of regulatory proteins and shows high homology to regulatory protein OtcG from OTC biosynthesis, identified by Lešnik et al. [13]. Close homologs of ChdC are found in oxytetracycline, dactylocycline and chlortetracycline BGC, OtcG, DacT3 and CtcA, respectively are presented on Additional file 1: Figure S2. In OTC biosynthesis, OtcG has a conditionally positive regulatory role in OTC production. As demonstrated by Lesnik et al., *otcG* inactivation reduced the production of OTC by more than 40%, while its overexpression under a strong constitutive promoter did not yield any statistically significant change in the production of OTC [13]. Therefore, in the scope of this study, we mostly focused on the SARP transcription activator ChdB,

Finally, *chdR* encodes a putative integral membrane protein that is most probably responsible for the efflux of CHD from the cell and is probably regulated by another regulatory protein, the putative TetR family repressor protein ChdA [1]. Interestingly, despite the relatively high structural similarity between OTC and CHD structures, ChdR from CHD BGC does not share high homology with the OtrB exporter from OTC BGC, suggesting significant differences in mode of action and the structure of TCs and CHD. The resistance to CHD may have a significant impact on the production of antibiotic in heterologous host. Therefore, we intended to carry out in-trans over-expression of *chdR* gene, which is likely providing resistance to CHD and CD-CHD in *A. sulphurea* and thus, may also have an important role in the heterologous host.

### Overexpression of streptomyces antibiotic regulatory protein (SARP) chdB gene

We carried out the overexpression of an additional copy of SARPs *chdB* and *otcR* from *S. rimosus* [14] in native producer *A. sulphurea* as described in materials and methods. The selected genes were placed under expression of *ActIIORF4/PactI* activator/promotor system [25], resulting in pAB03otcR and pAB03-SARP (Additional file 1: Table S2). A ΦBT- based integrative vector pAB03 was used for in-trans integration of the selected genes.

**Fig. 4:**
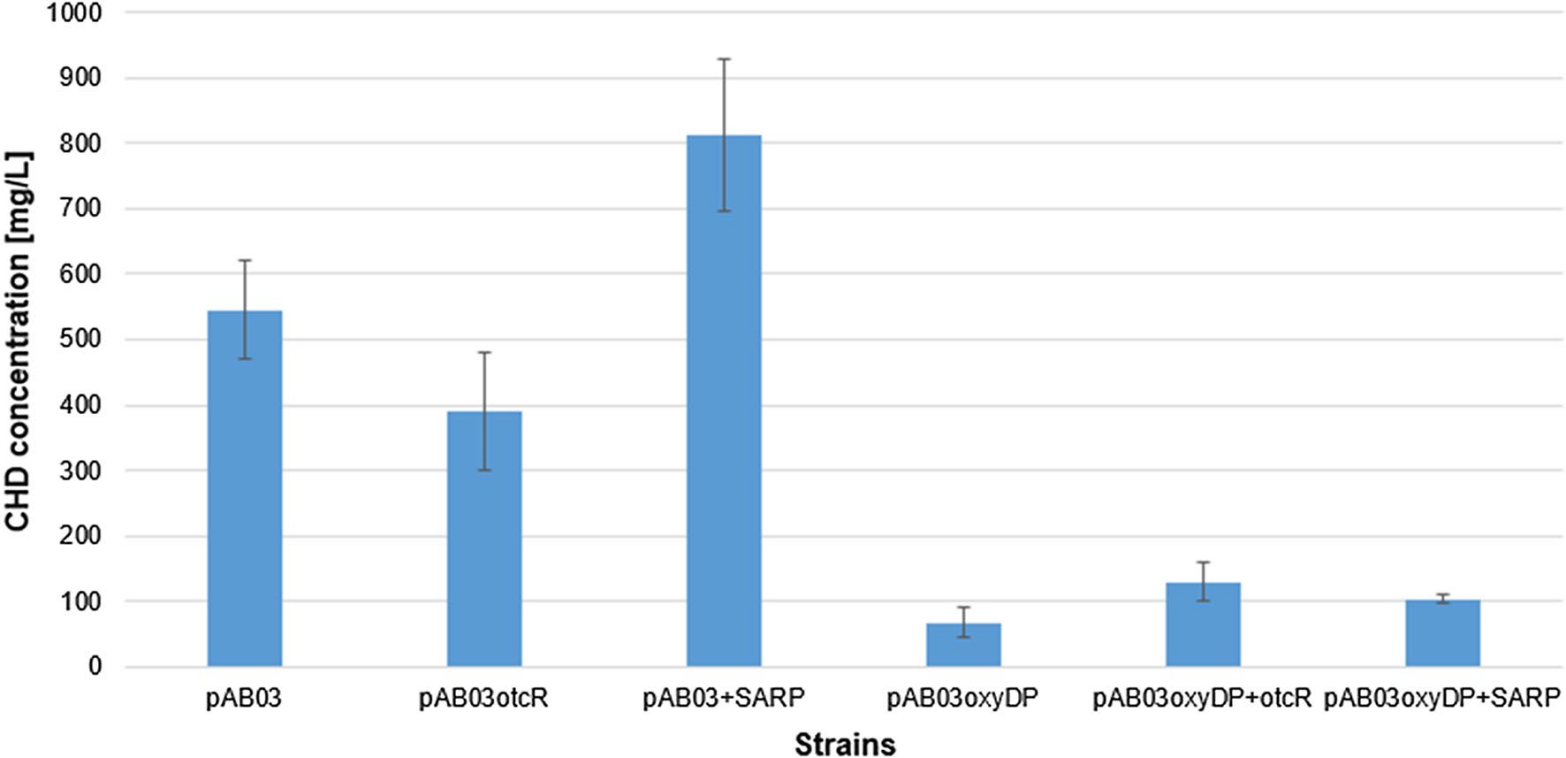
Overexpression of SARP homologues *otcR* from *S. rimosus* and *chdB* in native producer *A. sulphurea*

Overexpression of native SARP *chdB* clearly increased the yield of CHD and CDCHD for 1.5- to 1.9-fold, respectively (Additional file 1: Table S3). However, additional copy of SARP homologue of otcR from *S. rimosus* M4018, did not have significant positive effect on CHD production. In contrast, when *otcR* was overexpressed in the CDCHD-producing strain of *A. sulphurea*, there was clearly a positive effect on the production of target compound (Fig. 4). Thus, we can confirm that *chdB* is a positive regulatory protein in CHD biosynthesis.

### Cloning and heterologous expression of CHD BGC using replicative and integrative cosmids

After screening of the newly constructed *A. sulphurea* cosmid library based on the replicative conjugative cosmid vector pOJ456, a cosmid containing the entire CHD BGC was identified and designated as pOJ456-CHD12. Cosmid library construction and the subsequent cloning and heterologous expression procedures are described in the materials and methods section. The selected cosmid pOJ456-CHD12 was introduced into *S. albus* del14 [11] by conjugation in an attempt to heterologously express the CHD BGC. The model organism *S. albus* was selected as a heterologous host considering, this strain is significantly more amenable to genetic manipulations than the native producer *A. sulphurea*, which would help in faster development of new analogues. *S. albus* del14 strain, developed by Myronovskyi et al., was optimized even further by deletion of its native clusters encoding secondary metabolite biosynthetic pathways [11].

**Fig. 5:**
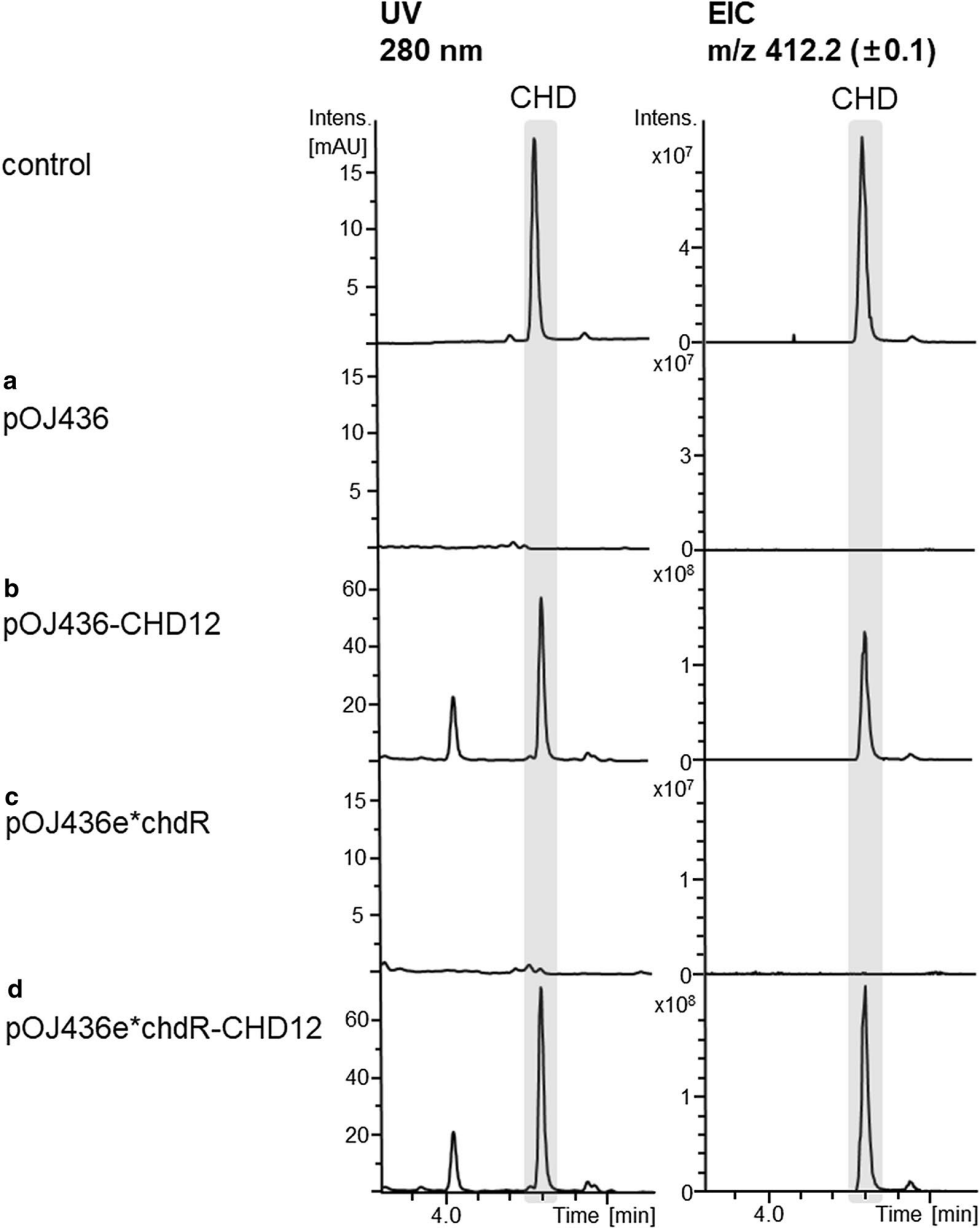
LC–MS analysis of culture extracts of *S. albus*: Chromosomally-integrated empty cosmids pOJ436 (**a**) or pOJ436e*chdR (**c**) in comparison to culture extracts of *S. albus* containing chromosomally-integrated cosmids carrying the entire CHD BGC (pOJ436-CHD12) (**b**) or CHD BGC with additional copy of efflux pump *chdR gene* (pOJ436e*chdR-CHD12) (**d**). UV chromatograms at detection wavelength of 280 nm and EIC for m/z 412 (± 0.5), which corresponds to CHD, are shown (chromatograms adapted from DataAnalysis (Bruker Daltonics, Bremen))

However, by applying liquid chromatography–mass spectroscopy (LC–MS) analysis no CHD could be detected in culture broth extracts of *S. albus* del14 carrying cosmid pOJ456-CHD12, which could be due to instability of the replicative cosmid or CHD self-resistance issues. Therefore, we transferred the entire CHD BGC into an integrative cosmid pOJ436, containing the ΦC31 integrase, to allow a stable integration of CHD BGC into the genome of *S. albus* del14. Indeed, heterologous expression of CHD BGC from chromosomally integrated cosmid pOJ436-CHD12 was successful and resulted in the production of CHD, reaching a yield of around 50 mg/L (Fig. 5, Table 2).

**Table 2:** Description of the *S. albus* del14 engineered strains for heterologous expression of CHD BGC, and corresponding product yields

Introduced cosmid	ChdR	OxyD	OxyP	CHD BGC	Product	Yield
pOJ456-CHD12				✓	/	/
pOJ436-CHD12				✓		Up to 50 mg/L
pOJ436-PermE*-chdR-CHD12	✓			✓		Up to 60 mg/L
pOJ436-PermE*-oxyDP-CHD12		✓	✓	✓	/	/
pOJ436-PermE*-oxyDPchdR-CHD12	✓	✓	✓	✓		~ 3 mg/L

### Attempt to increase the production yield of CHD in a heterologous host through overexpression of chdR efflux pump gene

Considering unusual mode of action of CHD [26], which is still not clearly understood, and in an attempt to increase yield of CHD by overcoming possible self-resistance issues during heterologous expression of CHD, we constructed a second integrative cosmid carrying entire CHD BGC with an additional copy of CHD efflux pump gene *chdR* under control of the strong constitutive promoter P_ermE*_ (construct pOJ436-PermE*-chdR-CHD12). However, the additional copy of efflux pump gene *chdR* did not lead to a significant increase of production yields of CHD in *S. albus* del14, reaching up to 60 mg/L (Fig. 5, Table 2).

### Production of CDCHD in the heterologous host

**Fig. 6:**
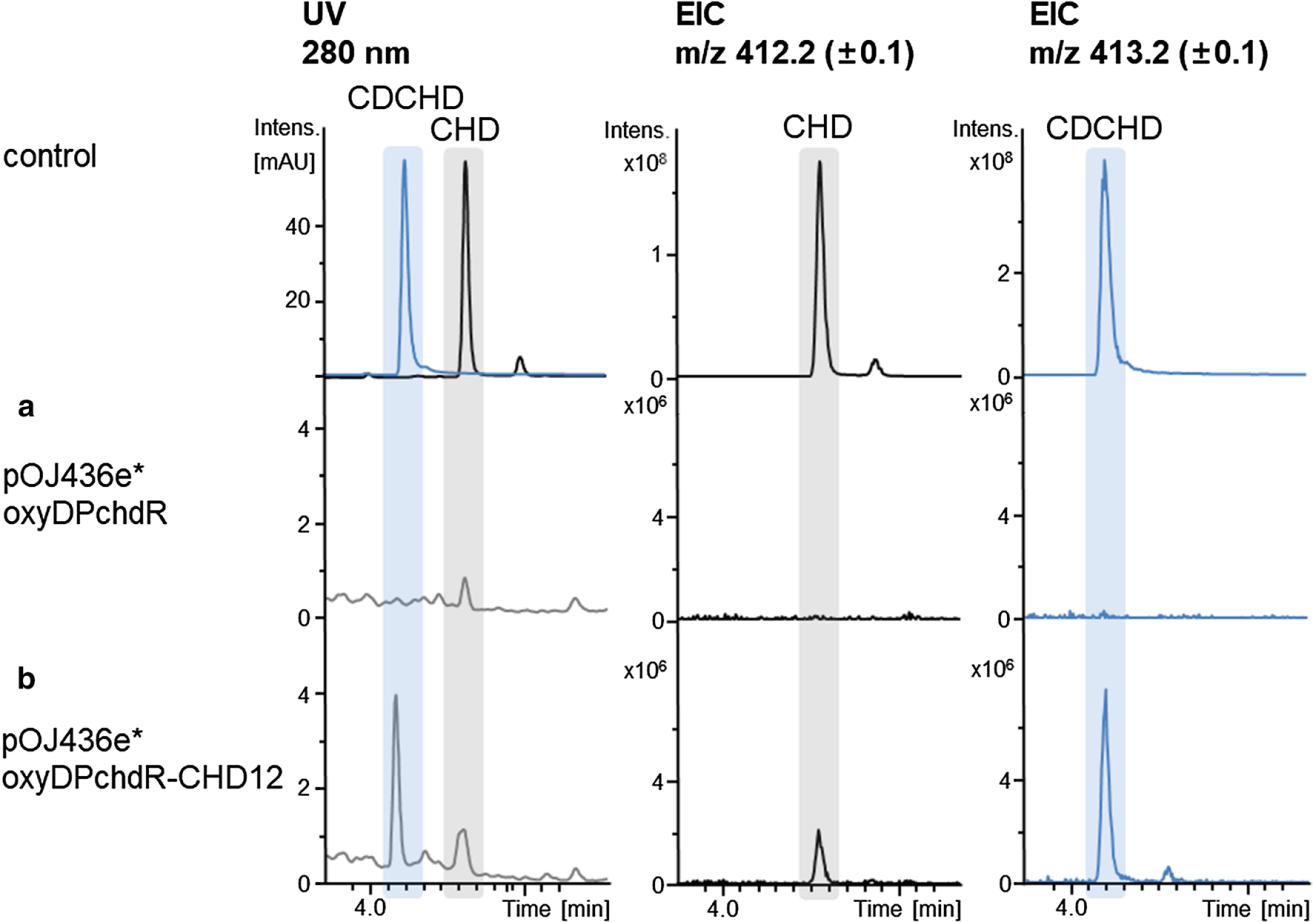
LC–MS analysis of culture extract of *S. albus*: Chromosomally-integrated empty cosmid pOJ436e*oxyDPchdR (**a**) in comparison with culture extract of *S. albus* with chromosomally-integrated cosmid carrying CHD BGC and *oxyD*, *oxyP* and *chdR* genes (pOJ436e*oxyDPchdR-CHD12) (**b**). UV chromatograms at detection wavelength of 280 nm and EICs for m/z 412 (± 0.5) and m/z 413 (± 0.5), which correspond to CHD and CDCHD, respectively, are shown (chromatograms adapted from DataAnalysis (Bruker Daltonics, Bremen))

We also aimed to produce CDCHD [8] as this compound exhibits superior antibacterial activity when compared to CHD, including activity against all Gram-negative pathogens of the ESKAPE panel [8]. To achieve this goal, we constructed two integrative cosmids; one carrying CHD BGC and *oxyDP* (genes for amidotransferase OxyD and acyltransferase OxyP from OTC BGC) and the second plasmid *oxyDPchdR* carrying genes for amidotransferase OxyD and acyltransferase OxyP from OTC BGC and an additional copy of efflux pump ChdR from CHD BGC, which were expressed under strong promoter P_ermE*_ (constructs pOJ436-PermE*-oxyDP-CHD12 and pOJ436-PermE*-oxyDPchdR-CHD12, respectively). Although at relatively low yield of approximately 3 mg/L, production of CDCHD was achieved in some *S. albus* del14 ex-conjugants with integrated pOJ436-PermE*-oxyDPchdR-CHD12 (Fig. 6, Table 2). Interestingly, production of CDCHD was not achieved in *S. albus* del14 ex-conjugants with integrated cosmid without the additional copy of efflux pump ChdR (construct pOJ436-PermE*-oxyDP-CHD12), suggesting lack of self-resistance by the engineered *S. albus* strains towards CDCHD.

## Conclusion

Although structurally similar to typical TCs such as OTC, CHD displays a different mode of action as antibacterial compound [27]. We have previously reported cloning of the respective BGC [1]. However, the entire genome sequence of producer strain *A. sulphurea*, which we have determined here, revealed an additional fragment of the CHD BGC containing a putative second ring cyclase gene homologue, which allowed us to explain previously puzzling biosynthetic steps of the early steps in CHD biosynthesis. Additionally, two more putative regulatory genes were identified, opening potential to aid in efforts to increase the production yield of CHD and other potential analogues of CHD such as CDCHD. In light of these new findings, we attempted to produce CHD in a genetically amenable heterologous host *S. albus*. The cloning of the entire CHD BGC and expression in *S. albus* on a single cosmid was successful. In addition, biosynthetic genes *oxyD* and *oxyP* from OTC BGC, involved in the supply of malonamate starter unit, were added to the cosmid carrying entire CHD BGC, to ensure production of the more potent CHD analogue, CDCHD, which is currently showing promising activity in preclinical evaluation [10].

When considering structure–activity correlation, the basic TC backbone of CHD contains two aromatized rings (D and C) in contrast to the OTC structure, which only has the first ring (D) aromatized. This seemingly small difference in the structure of the basic TC backbone however, most likely has a profound influence on the planarity, where CHD with two rings aromatized bears a more planar structure. It is also important to consider that there are significant differences in CHD and OTC BGCs, which lead to different moieties being incorporated into the basic TC scaffold. These include enzymes catalyzing priming steps, and late biosynthetic enzymes involved in methylation, addition of amino and hydroxyl groups at C9, C4, C6 and C5, respectively. C4 transamination, which occurs in opposite stereochemistry in both compound classes, can be considered as a branching point between CHD and TC biosynthesis (Fig. 3). At this point, CHD and OTC pathways split towards typical and atypical TCs, such as OTC and CHD biosynthesis, respectively (Fig. 3). Absence of C6 hydroxylation, di-methylation of the C4 amino group and presence of additional C9 methylation in CHD biosynthesis may also result in a more planar structure of CHD, hence contributing to its potent activity and bactericidal mode of action (in contrast to bacteriostatic TCs acting on ribosomes).

Studies comparing the BGCs of CHD and OTC also revealed some evolutionary aspects of the development of type II PKS enzyme complexes (Fig. 2). Interestingly, when analyzing the entire CHD BGC from *A. sulphurea*, and comparing it to the other type II PKS, we observed that some areas in the CHD BGC encoding the C-9 methyltransferase *chdMII* and the glycosyl-transferase *chdGIV*, (Fig. 2) do show high homology to aureolic acids BGCs, such as those encoding mithramycin and chromomycin. In addition, we have also identified four genes, including the *chdN* carbon-4 aminotransferase and the exporter *chdR* (Fig. 2), which do not show any significant homology to other genes found in type-II PKS BGC. Although CHD seems structurally very similar to typical TCs, these genes make CHD distinct in structural properties and particularly in biological activity.

In this study we identified additional essential genes for CHD biosynthesis, which was confirmed by their heterologous expression in *S. albus* as heterologous host. The corrected nucleotide sequence of the CHD BGC therefore contains all gene products required for the production of CHD. The heterologous system is significantly more amenable to genetic manipulation, thus opening new possibilities towards production of novel and potent TC analogues exhibiting a new mode of action.

## Methods

### Bacterial strains and culture conditions

*Amycolatopsis sulphurea* NRRL 2822 (ARS Culture Collection) was used for production of chelocardin (CHD) and as a source of DNA and for microbiological manipulations. *Streptomyces rimosus* M4018 [28] was used as a source of DNA for cloning of genes from oxytetracycline (OTC) biosynthetic gene cluster (BGC). *Streptomyces albus* del14 [11] was used for heterologous expression of CHD BGC. *Escherichia coli* DH10β (Invitrogen) was used for standard cloning procedures [29], and *E. coli* GB2006 (Gene Bridges) for preparation of *A. sulphurea* cosmid library. *E. coli* ET12567 [30] carrying pUZ8002 plasmid [31] was used as a donor strain for intergeneric conjugation with *S. albus* del14 (Additional file 1: Table S1). Soya mannitol (MS) agar and tryptone soy broth (TSB) [32] were used as agar sporulation medium and for cultivation of actinomycetes in liquid medium, respectively. For heterologous production of CHD and CHD analogues, *S. albus* was cultivated in TSB seed medium and four different production media: CH-F2, DNPM (4% dextrin, 0.75% soytone, 0.5% baking yeasts, 2.1% MOPS, pH 6.8 [33]), NL5Y (0.1% NaCl, 0.1% KH_2_PO_4_, 0.05% MgSO_4_ × 7H_2_O, 2.5% glycerol, 0.584% l-glutamine, 0.2% trace elements solution, 1% yeast extract, pH 7.3 [34], and SG1 (2% glucose, 0.5% yeast extract, 1% soytone, 0.2% CaCO_3_, pH 7.2 [35]). Cultivations were performed in Falcon tubes at 30 °C on a rotary shaker at 220 rpm for 36 h in seed medium with 5% (v/v) used to inoculate production media and cultivated for further 7 days under the same conditions. For intergeneric conjugation between *S. albus* and *E. coli* MS medium, supplemented with 10 mM MgCl_2_ was used. Apramycin (50 µg/mL) together with nalidixic acid (25 µg/mL) was used for selection of *S. albus* exconjugants on MS. For selection of *E. coli* transformants, Apr (50 µg/mL), Kan (25 µg/mL) or chloramphenicol (Cm; 10 µg/mL) were added into LB medium.

### Overexpression of SARPs in A. sulphurea

Genes *otcR* from *S. rimosus* M4018 and *chdB* from *A. sulphurea* were PCR amplified (Additional file 1: Table S2) using otcR-Fw, OtcR-Rv and SARP-Fw, SARP-Rv primers, respectively. The PCR amplified DNA fragments were digested with *Nde*I and *Xba*I and separately cloned into pAB03, downstream of the *actI* promoter under control of the *ActIIORF4/PactI* activator/promotor system [25], resulting in pAB03otcR and pAB03-SARP (Additional file 1: Table S2), respectively. When the DNA fragments were intended for cloning into pABoxyDP plasmid, they were digested only with *Xba*I, which retained additional RBS sequence in front of the gene, yielding pAB03oxyDPotcR and pAB03oxyDP-SARP (Additional file 1: Table S2). Plasmids were used to transform *E. coli* SCS110 and together with pAB03 introduced into *A. sulphurea* via direct transformation of mycelium [36]. A ΦBT- based integrative pAB03 vector stably integrates into the chromosome of *A: sulphurea*. S27M plates were overlaid with Apr after 16-h incubation. Each transformant colony was further repatched onto MS agar containing Apr and incubated for 10 days. Transformants were inoculated in CHD-V medium followed by CHD-F2 medium, as described above. CHD and CDCHD were extracted from production broth and production yields were measured using LC–MS.

### DNA isolation and manipulation

Isolation and manipulation of DNA in *E. coli* were carried out according to standard protocols. [29, 32] Cosmids were introduced into *S. albus* del14 via conjugation [32]

### Sequencing of genomic DNA

Salting out procedure [32] was used to isolate genomic DNA from *A. sulphurea* which was submitted for sequencing with Illumina technology to Seq-IT (Kaiserslautern, Germany). Two separate sequencing libraries were prepared (paired-end, mate-pair) which were used for cluster generation on a single lane of MiSeq instrument (50%/50% fill rate). Obtained raw sequencing read data was then assembled with the help of abyss-pe 1.3.6 software [37]. The estimated genome size of *A. sulphurea* NRRL 2822 is 7.0 mbp and was contained in 126 contigs on 8 scaffolds.

### Preparation of A. sulphurea cosmid library

Genomic DNA was partially digested with *Sau3A*I and the DNA fragments of approximate size 35–40 kb were ligated into the *BamH*I site of replicative conjugative cosmid vector pOJ456, a modified version of the pOJ436 vector [38], where 2.5 kb ΦC31 integrase cassette was excised with *Hind*III (overhangs were filled in with Klenow polymerase) and replaced with 2.5 kb pSG5 replication cassette excised with *Eco81*I and *Sph*I (overhangs were filled in with Klenow polymerase) from medium copy number vector pKC1139 [38]. The ligated DNA was packaged into phage particles (Gigapack III Gold Packaging kit, Agilent Technologies) and introduced into *E. coli* GB2006.

### Identification and sequencing of cosmid carrying CHD BGC

The cosmid library was screened by combining all 3400 colonies and streaking the mixture onto LB agar plates supplemented with 3 µg/mL of CHD to select for CHD-resistant single colonies expressing ChdR efflux pump encoded in the CHD BGC. 18 positive clones were selected to isolate cosmid DNA and additional PCR screening was carried out using the primer pairs CobU1/CobU2 and glu1/glu2 (Additional file 1: Table S2), designed to anneal to the flanking regions of CHD BGC. Based on the PCR screen, two cosmids were selected for complete sequencing by Illumina sequencing, resulting in confirmation of cosmid pOJ456CHD12, carrying the complete CHD BGC, whose correct and complete sequence was also identified from genomic DNA sequence of *A. sulphurea* NRRL 2822. The GenBank Accession Number for the revised CHD BGC is KC870000.

### Construction of different versions of cosmids carrying CHD BGC

34 kb DNA fragment encoding CHD BGC from pOJ456CHD12 was cloned via *Spe*I and *Xba*I into integrative conjugative cosmids pOJ436, pOJ436e*chdR, pOJ436e*oxyDP, or pOJ436e*oxyDPchdR, resulting in pOJ436CHD12, pOJ436e*chdRCHD12, pOJ436e*oxyDPCHD12, and pOJ436e*oxyDPchdRCHD12, respectively. pOJ436e*chdR, pOJ436e*oxyDP, or pOJ436e*oxyDPchdR were constructed from pOJ436 by introducing 1.8 kb, 3.2 kb and 4.7 kb fragments, carrying *chdR*, *oxyDP* and *oxyDPchdR* genes, respectively, all under the control of P_ermE*_ promoter. Fragments were excised with *Ecl136*II (overhang was filled in with Klenow polymerase) and *Xba*I from plasmids pAB03e*chdR, pAB03e*oxyDP and pAB03e*oxyDPchdR, respectively, and used to replace the 1.9 kb fragment in pOJ436, excised with *Nru*I (overhang was filled in with Klenow polymerase) and *Xba*I. pAB03e*chdR was constructed by cloning 1.5 kb *chdR* gene, amplified by PCR using primers chdRF and chdRR (Additional file 1: Table S2), digested with *Nde*I and *Xba*I and ligated into pAB03e* (pAB03 vector with P_ermE*_ promoter instead of actII-ORF4/P_actI_ activator/promoter system). pAB03e*oxyD was constructed by cloning *oxyD* gene, excised from pAB03oxyD [1] with *Nde*I and *Xba*I and ligated into pAB03e*. pAB03e*oxyDP was constructed by cloning *oxyP* gene, excised from pAB03oxyDP [1] with *Xba*I, into *Xba*I site of pAB03e*oxyD downstream of *oxyD* gene. pAB03e*oxyDPchdR was constructed by cloning *chdR* gene, excised from pAB03e*chdR with *Cla*I and *Hind*III (overhangs were filled in with Klenow polymerase), into *Xba*I (overhangs were filled in with Klenow polymerase) site of pAB03e*oxyDP downstream of *oxyP* gene.

### Heterologous expression of CHD BGC

Cosmids carrying the CHD BGC, pOJ456CHD12, pOJ436CHD12, pOJ436e*chdRCHD12, pOJ436e*oxyDPCHD12 and pOJ436e*oxyDPchdRCHD12, and empty control cosmids, pOJ456, pOJ436, pOJ436e*chdR, pOJ436e*oxyDP and pOJ436e*oxyDPchdR were transformed into *E. coli* ET12567 [30] carrying PUZ8002, which was then used as donor strain for intergeneric conjugation with *S. albus* del14. MS plates supplemented with 10 mM MgCl_2_ were overlaid with Apr and nalidixic acid after overnight incubation. Each exconjugant was further repatched onto MS agar containing Apr (50 µg/mL) and nalidixic acid (25 µg/mL), followed by cultivation in liquid media as described above. Culture broths were extracted and analysed by liquid chromatography–mass spectroscopy (LC–MS) to check for production of CHD or CHD analogues.

### Liquid chromatography–mass spectroscopy analysis

To measure the yield of CHD and 2-carboxamido-2-deacetyl-chelocardin (CDCHD), *S. albus* culture broths were acidified to pH 1–2 with 50% TFA, followed by extraction with 2 V of MeOH. The extract was centrifuged and analyzed by LC–MS. All measurements were performed on a Dionex Ultimate 3000 LC system using a Luna C-18 [2] HST, 100 × 2.0 mm, 2.5 µm column (Phenomenex). Separation of 1 µl sample was achieved by a linear gradient from (A) H_2_O + 0.1% FA to (B) ACN + 0.1% FA at a flow rate of 500 µl/min and 45 °C. The gradient was initiated by a 0.5 min isocratic step at 5% B, followed by an increase to 95% B in 9 min to end up with a 1.5 min step at 95% B before re-equilibration with initial conditions. To achieve better separation of peaks, gradient was extended to 18 min. UV spectra were recorded by a DAD in the range from 200 to 600 nm. The MS measurement was carried on an amaZon speed mass spectrometer (Bruker Daltonics, Bremen) using the standard ESI source. Mass spectra were acquired in centroid mode ranging from 200–2000 m/z in positive ionization mode.

## Supplementary Information

**Additional file 1.** **Table S1. **Bacterial strains and plasmids used in this study^*a*^. **Table S2. **Sequences of the oligonucleotide primers for PCR experiments used in this study^a^. **Table S3.** Overexpression of SARPs *otcR* and *chdB* in *A. sulphurea* WT strain. **Figure S1.** Protein alignment of ChdB with closest homologs present in BGC encoding type II PKS. Close homologs of *Streptomyces* antibiotic regulatory protein (SARP) from oxytetracycline, SF2575, dactylocycline and chlorotetracycline BGC, OtcR, SsfT1, DacT1, CtcB, respectively are presented. Gray colour denotes the similarity of the conserved amino acid residues. The OmpR/PhoB-type DNA-binding domain with a typical fold of the helix-turn-helix is marked with ––, whereas the conserved DNA-binding sites are marked with *. Additional DNA-binding domain marked with ~ contains three tetratricopeptide repeats (TPRs) and two C-terminal helices. The TPR motif generates a right-handed helical structure with an amphipathic channel that is thought to accommodate an alpha-helix of a target protein. **Figure S2.** Protein alignment of ChdC with closest homologs present in BGC encoding type II PKS. Close homologs from oxytetracycline, dactylocycline and chlorotetracycline BGC, OtcG, DacT3 and CtcA, respectively are presented. Gray colour denotes the similarity of the conserved amino acid residues. The Sigma-70 domain involved in binding to the -35 promoter element via a helix-turn-helix motif is marked with ~, the signal receiver domain is marked –––, the TTA codon is marked with red rectangle, the phosphorylation site is marked with +, and the dimerization interface is marked with ***. (7)

## Data Availability

All data generated or analysed during this study are included in this published article and its additional information files. We will introduce corrections into the current CHD gene cluster nucleotide sequence into GenBank Accession Number KC870000 once paper is at the final stages of reviewing.
