# Supplementary material for: Heterologous expression of the atypical tetracycline chelocardin reveals the full set of genes required for its biosynthesis
Source: Microb Cell Fact. 2020 Dec 19;19:230. doi: 10.1186/s12934-020-01495-x (PMC7749508; doi:10.1186/s12934-020-01495-x)
Supplement: Supplementary file 1 — Additional file 1. Table S1. Bacterial strains and plasmids used in this studya. Table S2. Sequences of the oligonucleotide primers for PCR experiments used in this studya. Table S3. Overexpression of SARPs otcR and chdB in A. sulphurea WT strain. Figure S1. Protein alignment of ChdB with closest homologs present in BGC encoding type II PKS. Close homologs of Streptomyces antibiotic regulatory protein (SARP) from oxytetracycline, SF2575, dactylocycline and chlorotetracycline BGC, OtcR, SsfT1, DacT1, CtcB, respectively are presented. Gray colour denotes the similarity of the conserved amino acid residues. The OmpR/PhoB-type DNA-binding domain with a typical fold of the helix-turn-helix is marked with ––, whereas the conserved DNA-binding sites are marked with *. Additional DNA-binding domain marked with ~ contains three tetratricopeptide repeats (TPRs) and two C-terminal helices. The TPR motif generates a right-handed helical structure with an amphipathic channel that is thought to accommodate an alpha-helix of a target protein. Figure S2. Protein alignment of ChdC with closest homologs present in BGC encoding type II PKS. Close homologs from oxytetracycline, dactylocycline and chlorotetracycline BGC, OtcG, DacT3 and CtcA, respectively are presented. Gray colour denotes the similarity of the conserved amino acid residues. The Sigma-70 domain involved in binding to the -35 promoter element via a helix-turn-helix motif is marked with ~, the signal receiver domain is marked –––, the TTA codon is marked with red rectangle, the phosphorylation site is marked with +, and the dimerization interface is marked with ***. (7) [file 12934_2020_1495_MOESM1_ESM.doc]

**Additional Information for:**

**Heterologous expression of the atypical tetracycline chelocardin reveals the full set of genes required for its biosynthesis**

Tadeja Lukežič^[a,b]#^, Špela Pikl^[c]^, Nestor Zaburannyi^[a,b]^, Maja Remškar^[a,b]^, Hrvoje Petković^[c]^*, Rolf Müller^[a,b]^*

From the ^[a]^ Department of Microbial Natural Products, Helmholtz-Institute for Pharmaceutical Research Saarland (HIPS), Helmholtz Centre for Infection Research (HZI) and Department of Pharmacy, Saarland University, Campus E8.1, 66123 Saarbrücken, Germany; ^[b]^ German Centre for Infection Research (DZIF), Partner site Braunschweig, Germany; the ^[c]^ Department of Food Science and Technology, Biotechnical Faculty, University of Ljubljana, Jamnikarjeva 101, 1000 Ljubljana, Slovenia

Running title: *Biosynthesis of atypical tetracyclines: chelocardin*

^#^ Present address: National Institute of Biology, Večna pot 111, 1000 Ljubljana, Slovenia

* To whom correspondence should be addressed: Rolf Müller, Helmholtz-Institute for Pharmaceutical Research Saarland (HIPS), Helmholtz Centre for Infection Research (HZI) and Department of Pharmacy, Saarland University, Campus E8.1, 66123 Saarbrücken, Germany; tel: +4968130270201; fax: +4968130270202; E-mail: [rolf.mueller@helmholtz-hips.de](mailto:rolf.mueller@helmholtz-hips.de); and Hrvoje Petković, Biotechnical faculty, University of Ljubljana, Jamnikarjeva 101, 1000, Ljubljana, Slovenia; tel: +38640488498; E-mail: [hrvoje.petkovic@bf.uni-lj.si](mailto:hrvoje.petkovic@bf.uni-lj.si)

**Table S1**

**Bacterial strains and plasmids used in this study*^a^***

| **Strain or plasmid** | **Relevant characteristics** | **Reference or source** |
| --- | --- | --- |
| *Escherichia coli* | | |
| DH10β | F^-^ *endA*1 *recA*1 *galE*15 *galK*16 *upG rpsL* Δ*lacX*74 Φ80*lacZ*ΔM15 *araD*139 Δ(*ara*-*leu*)7697 *mcrA* Δ(*mrr*-*hsdRMS*-*mcrBC*) λ^-^ | Invitrogen |
| ET12567 | F^-^ *dam*13::Tn*9, dcm*6, *hsdM*, *hsdR*, *recF*143::Tn*1I, galK*2, *galT*22, *ara*14, *lacY*1, *xyl*5, *leuB*6, *thi*1, *tonA*31, *rpsL*136, *hisG*4, *tsx*78, *mtl*1 *glnV*44 | (1) |
| GB2006 | δM109 *rpsL*- ∆*rfuA* | Gene Bridges |
| *Amycolatopsis sulphurea* | | |
| NRRL 2822 | WT producer of CHD | ARS Culture Collection |
| *Streptomyces rimosus* M4018 | Producer of OTC | (2) |
| *Streptomyces albus* del14 | host strain for heterologous expression | (3) |
| Plasmids |  |  |
| pAB03 | pSET152-derived, containing ΦBT, Apr^r^ | (4) |
| pAB03oxyDP | *oxyD* and *oxyP* cloned into pAB03 | (5) |
| pAB03otcR | *otcR* cloned into pAB03 | This study |
| pAB03oxyDP-otcR | *oxyD*, *oxyP* and *otcR* cloned into pAB03 | This study |
| pAB03SARP | *SARP* cloned into pAB03 | This study |
| pAB03oxyDP-SARP | *oxyD*, *oxyP* and *SARP* cloned into pAB03 | This study |
| pOJ436 | pSET152-derived cosmid, containing ΦC31, Apr^r^ | (6) |
| pKC1139 | bifunctional *oriT* RK2 vector, pSG5 ori, Apr^r^ | (6) |
| pOJ456 | pOJ436-derived cosmid, ΦC31 integrase cassette replaced with pSG5 replication cassette, Apr^r^ | This study |
| pOJ456CHD12 | pOJ456 cosmid carrying CHD BGC | This study |
| pAB03e* | pAB03 vector with P_ermE*_ promoter instead of actII-ORF4/PactI activator/promoter system | Acies Bio |
| pAB03e*chdR | pAB03e* carrying *chdR* gene | This study |
| pAB03e*oxyD | pAB03e* carrying *oxyD* gene | This study |
| pAB03e*oxyDP | pAB03e* carrying *oxyD* and *oxyP* genes | This study |
| pAB03e*oxyDPchdR | pAB03e* carrying *oxyD*, *oxyP* and *chdR* genes | This study |
| pOJ436e*chdR | pOJ436 carrying a 1.8 kb fragment from pAB03e*chdR containing *chdR* gene under the control of P_ermE*_ promoter | This study |
| pOJ436e*oxyDP | pOJ436 carrying a 3.2 kb fragment from pAB03e*oxyDP containing *oxyD* and *oxyP* genes under the control of P_ermE*_ promoter | This study |
| pOJ436e*oxyDPchdR | pOJ436 carrying a 4.7 kb fragment from pAB03e*oxyDPchdR containing *oxyD*, *oxyP* and *chdR* genes under the control of P_ermE*_ promoter | This study |
| pOJ436CHD12 | pOJ436 carrying CHD BGC | This study |
| pOJ436e*chdRCHD12 | pOJ436e*chdR carrying also CHD BGC | This study |
| pOJ436e*oxyDPCHD12 | pOJ436e*oxyDP carrying also CHD BGC | This study |
| pOJ436e*oxyDPchdRCHD12 | pOJ436e*oxyDPchdR carrying also CHD BGC | This study |
| *^a^* Apr^r^, apramycin resistant; Kan^r^, kanamycin resistant. | | |

**Table S2**

**Sequences of the oligonucleotide primers for PCR experiments used in this study*^a^***

| **Primers** | **Sequence** |
| --- | --- |
| CobU1 | 5'-TCCTCACTGCAGGTCGAGTACC-3' |
| CobU2 | 5'-CGGGAAGTCGCGGTATGC-3' |
| glu1 | 5'-CGCGCTGGTCAAAGTCTACG -3' |
| glu2 | 5'-CTGGACGCCTCGCCGTAC-3' |
| chdRF | 5'-TATATACATATGAAGGACAATCTCGCGAGA-3' |
| chdRR | 5'-TATATATCTAGAGGACCTCCGCATCAGGC-3' |
| otcR-Fw | ATATATTCTAGATCCGATTAATTAAGGAGGACACATATGGACTTCAAGGCACTCGGC |
| otcR-Rv | TATATATCTAGATTCAAGACGCCGACCTCAACAC |
| SARP-Fw | ATATATTCTAGATCCGATTAATTAAGGAGGACACATATGAAATTCAACCTGCTTGGTCCG |
| SARP-Rv | TATATATCTAGATTCACACATGCGCGGGTG |
| *^a^* Restriction sites are underlined. | |

**Table S3**

**Overexpression of SARPs *otcR* and *chdB* in *A. sulphurea*** WT strain

| **Strain** | **(CD)CHD concentration [mg/L]** | **Comment** |
| --- | --- | --- |
| WT/pAB03 | 546 ± 76 |  |
| WT/pAB03otcR | 390 ± 89 | 0.7× CHD production |
| WT/pAB03+SARP | 812 ± 117 | 1.5× CHD production |
| WT/ pAB03oxyDP | 68 ± 23 |  |
| WT/ pAB03oxyDP+otcR | 130 ± 29 | 1.9× CDCHD production |
| WT/ pAB03oxyDP+SARP | 103 ± 7 | 1.5× CDCHD production |

**
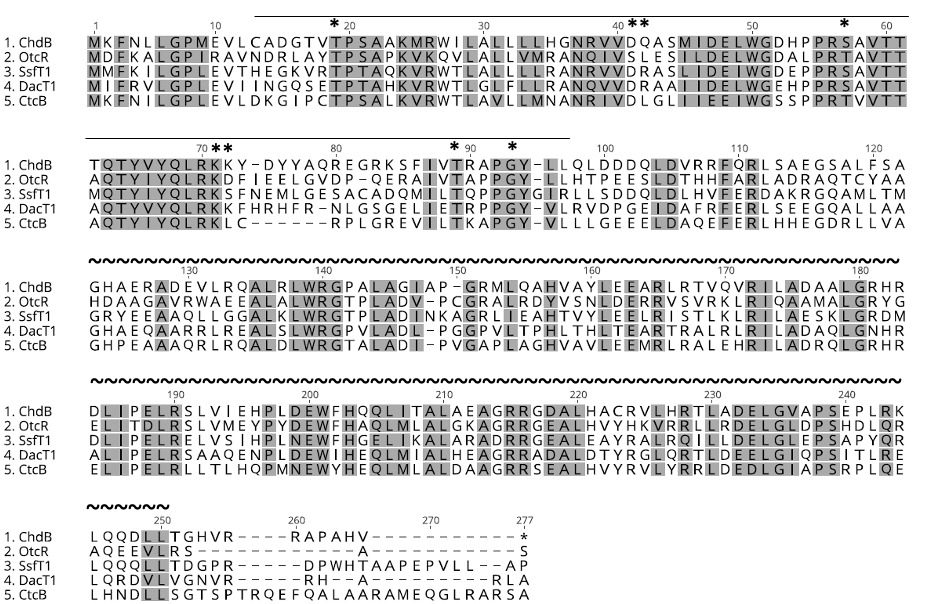
**

**Figure S1. Protein alignment of ChdB with closest homologs present in BGC encoding type II PKS.** Close homologs of *Streptomyces* antibiotic regulatory protein (SARP) from oxytetracycline, SF2575, dactylocycline and chlorotetracycline BGC, OtcR, SsfT1, DacT1, CtcB, respectively are presented. Gray colour denotes the similarity of the conserved amino acid residues. The OmpR/PhoB-type DNA-binding domain with a typical fold of the helix-turn-helix is marked with ––, whereas the conserved DNA-binding sites are marked with *. Additional DNA-binding domain marked with ~ contains three tetratricopeptide repeats (TPRs) and two C-terminal helices. The TPR motif generates a right-handed helical structure with an amphipathic channel that is thought to accommodate an alpha-helix of a target protein.

**
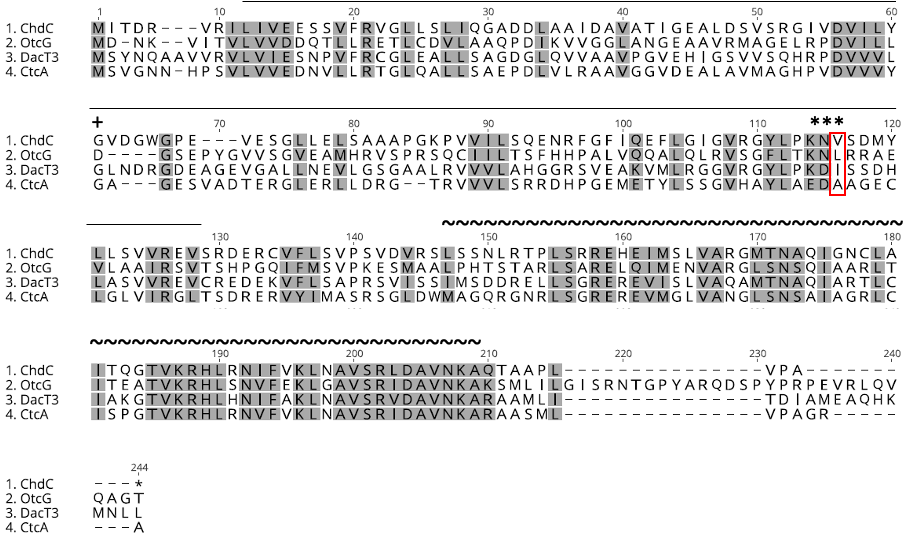
**

**Figure S2. Protein alignment of ChdC with closest homologs present in BGC encoding type II PKS.** Close homologs from oxytetracycline, dactylocycline and chlorotetracycline BGC, OtcG, DacT3 and CtcA, respectively are presented. Gray colour denotes the similarity of the conserved amino acid residues. The Sigma-70 domain involved in binding to the -35 promoter element via a helix-turn-helix motif is marked with ~, the signal receiver domain is marked –––, the TTA codon is marked with red rectangle, the phosphorylation site is marked with +, and the dimerization interface is marked with ***. (7)

**References**

1. MacNeil DJ, Gewain KM, Ruby CL, Dezeny G, Gibbons PH, MacNeil T. Analysis of Streptomyces avermitilis genes required for avermectin biosynthesis utilizing a novel integration vector. Gene. 1992;111(1):61–8.

2. Rhodes PM, Hunter IS, Friend EJ, Warren M. Recombinant DNA methods for the oxytetracycline producer Streptomyces rimosus. Biochem Soc Trans. 1984;12(4):586–7.

3. Myronovskyi M, Rosenkränzer B, Nadmid S, Pujic P, Normand P, Luzhetskyy A. Generation of a cluster-free Streptomyces albus chassis strains for improved heterologous expression of secondary metabolite clusters. Metab Eng. 2018;49:316–24.

4. Lukezic T, Lesnik U, Podgorsek A, Horvat J, Polak T, Sala M, et al. Identification of the chelocardin biosynthetic gene cluster from Amycolatopsis sulphurea: a platform for producing novel tetracycline antibiotics. Microbiology. 2013;159(Pt 12):2524–32.

5. Lešnik U, Lukežič T, Podgoršek A, Horvat J, Polak T, Šala M, et al. Construction of a new class of tetracycline lead structures with potent antibacterial activity through biosynthetic engineering. Angew Chemie - Int Ed. 2015;54(13):3937–40.

6. Bierman M, Logan R, O’Brien K, Seno ET, Rao RN, Schoner BE. Plasmid cloning vectors for the conjugal transfer of DNA from Escherichia coli to Streptomyces spp. Gene. 1992;116(1):43–9.

7. Lešnik U, Gormand A, Magdevska V, Fujs Š, Raspor P, Hunter I, et al. Regulatory elements in tetracycline-encoding gene clusters: The otcG gene positively regulates the production of oxytetracycline in Streptomyces rimosus. Food Technol Biotechnol. 2009;47(3):323–30.
